# Supplementary material for: Impaired BDNF-TrkB trafficking and signalling in Down syndrome basal forebrain neurons
Source: Cell Death Dis. 2026 Feb 11;17(1):214. doi: 10.1038/s41419-026-08464-z (PMC12921309; doi:10.1038/s41419-026-08464-z)
Supplement: Supplementary file 1 — Supplementary Figure Legends [file 41419_2026_8464_MOESM1_ESM.docx]

**Supplemental Material**

**Supplementary Figure 1**

(A) Representative confocal images of total Rab5 expression in the axon of WT and Dp1Tyb BFNs. Scale bar = 10 μm.

(B) No significant difference in the intensity of total Rab5 expression in the axon of WT and Dp1Tyb BFNs (Welch’s test, p= 0.3166). However, F test comparing variances suggests that Rab5 expression is significantly more variable in Dp1Tyb neurons compared to WT (p < 0.0001). *N* = 6 biological replicates.

(C) Representative confocal images of SH-SY5Y cells transfected with constitutively-active Rab5^Q79L^ and dominant-negative Rab5^N133I^ mutants, then incubated with GST-Rab5BD and stained for GST. Scale bar = 10 µm.

(D) SH-SY5Y cells were incubated with GST alone to identify any background staining resulting from GST binding. Scale bar = 10 µm.

(E) SH-SY5Y cells transfected with Rab5^Q79L^ showed increased binding of GST-Rab5BD compared to untransfected cells and cells transfected with Rab5^N133I^ (2-way ANOVA with Tukey’s multiple comparisons; ****, p<0.0001). GST bound significantly less than GST-Rab5BD indicating the specificity of this fusion protein (2-way ANOVA with Tukey’s multiple comparisons; Rab5^Q79L^; ****, p<0.0001; Rab5^N133I^; *, p=0.0153). *N*= 3 individual transfections.

For all graphs, different coloured points represent separate *N*s. Bars represent mean ± SEM.

**Supplementary Figure 2**

(A) Representative confocal images of TrkB expression at DIV14. Scale bar = 10 μm.

(B) Line graph showing average TrkB intensity in the (i) soma and (ii) neurites over time from DIV2 to DIV14 in Dp1Tyb and WT BFNs. Values were normalised to the average of WT DIV2+DIV4. There was an overall effect of DIV on TrkB expression in the soma and the neurites (2-way ANOVA with Tukey’s multiple comparisons; soma; ****, p<0.0001, neurites; *** p=0.0003 respectively). There was no effect of genotype (2-way ANOVA with Tukey’s multiple comparisons, soma p=0.0809, neurites p=0.3019). Shaded areas represent SEM. *N* =3 biological replicates.

(C) Representative confocal images of surface TrkB expression in WT and Dp1Tyb BFNs. Scale bar = 10 μm.

(D) No significant difference in surface TrkB expression levels in WT vs Dp1Tyb BFNs (unpaired Student’s *t* test, p= 0.7076). *N* = 4 biological replicates.

(E) Western blot showing TrkB-FL and TrkB T1 in WT and Dp1Tyb basal forebrain cultures at DIV14.

(F) No significant difference in the expression of (i) TrkB-FL or (ii) TrkB-T1 (iii) or TrkB-FL/TrkB-T1 ratio between WT and Dp1Tyb basal forebrain cultures. Values were normalised to total protein. *N*= 3 biological replicates.

For all graphs, different coloured points represent separate *N*s. Bars represent mean ± SEM.

**Supplementary Figure 3**

(A) Representative confocal images of HcT/TrkB colocalization in the axon ± BDNF treatment. Scale bar = 10 μm. White arrowheads indicate double-positive HcT/TrkB puncta in the axon.

(B) BDNF significantly increases the number of TrkB, HcT and double-positive HcT/TrkB puncta in the axon of WT neurons verifying that HcT signalling endosomes contain TrkB (2-way ANOVA with Bonferroni’s multiple comparisons, TrkB **** p<0.0001, HcT *** p= 0.0001, HcT/TrkB **** p<0.0001). *N* = 3 biological replicates.

(C) Signalling endosome transport speeds in WT and Dp1Tyb BFNs ± BDNF per axon. There was a significant effect of both BDNF and genotype on signalling endosome transport speeds (2-way ANOVA with Tukey’s multiple comparisons, BDNF; **** p<0.0001, genotype; * p= 0.0372).

(D) Percentage of pausing of signalling endosomes in WT and Dp1Tyb BFNs ± BDNF per axon. There was a significant effect of both BDNF and genotype on signalling endosome pausing (2-way ANOVA with Tukey’s multiple comparisons, BDNF; ****, p<0.0001, genotype; **** p<0.0001, interaction; ** p= 0.0068).

(*N* = 6 biological replicates, 5-8 axons imaged per condition).

(E, F) The number of signalling endosomes in not significantly altered in WT or Dp1Tyb BFNs following BDNF treatment (paired Student’s *t* test, WT p= 0.162, Dp1Tyb p= 0.195). *N* = 6 biological replicates.

(G) Representative confocal images of total Rab7 expression in the axon of WT and Dp1Tyb BFNs. Scale bar = 10 μm.

(H) No significant difference in total Rab7 intensity between WT and Dp1Tyb BFNs.

Different coloured points represent separate *N*s. Bars represent mean ± SEM.

**Supplementary Figure 4**

(A) Representative confocal images of pAKT(Ser473) staining in WT and Dp1Tyb neurons.

(B) Quantification of pAKT(Ser473) intensity. No significant effect of BDNF or genotype on the phosphorylation of AKT at Ser473 with any length of treatment was detected. *N* = 3 biological replicates, 5-10 cells per replicate.

(C) Western blots showing the levels of pAKT(Ser473), total AKT, pERK1/2 and total ERK1/2 after treatment of WT and Dp1Tyb BFCNs with 50 ng/mL BDNF for 0, 30 or 120 min. GAPDH was used as a loading control. Phosphoprotein intensities were normalized to total protein load.

(D) Quantification of AKT activation revealed a significant effect of both BDNF treatment and genotype on pAKT levels (2-way ANOVA with Tukey’s multiple comparisons, BDNF; *** p= 0.0002, genotype; ** p =0.0010. Multiple comparisons; ***, p=0.0005, *, p=0.0184).

(E) Quantification of ERK1/2 activation revealed a significant increase in the activation of ERK1/2 with BDNF treatment (2-way ANOVA; ** p= 0.0014), however there was no significant difference between the genotypes. *N* = 4 biological replicates.

(F, G) Increased pERK1/2 staining in non-neuronal cells surrounding the neurons in Dp1Tyb cultures compared to WT. This activation of ERK1/2 in non-neuronal cells appeared to increase with BDNF treatment suggesting it is TrkB-dependent.

Scale bar = 20 µm. Bars represent mean ± SEM. Coloured points represent individual *N*s.

**Supplementary Figure 5**

Western blot of cell lysates upon treatment with ERK1/2 inhibitor. WT and Dp1Tyb BFCN cultures were depleted of growth factors and pre-treated with 20 µM U0126 for 1 h prior to stimulating with 50 ng/mL BDNF for 30 min. U0126 reduced the level of BDNF-induced ERK1/2 phosphorylation. U0126 IC_50_ = 10-20 µM. (p) indicates loading control for pERK1/2 blot and (t) indicates the loading control for ERK1/2.
