# Supplementary material for: Impaired BDNF-TrkB trafficking and signalling in Down syndrome basal forebrain neurons
Source: Cell Death Dis. 2026 Feb 11;17(1):214. doi: 10.1038/s41419-026-08464-z (PMC12921309; doi:10.1038/s41419-026-08464-z)

Supplementary Figure 2. total TrkB

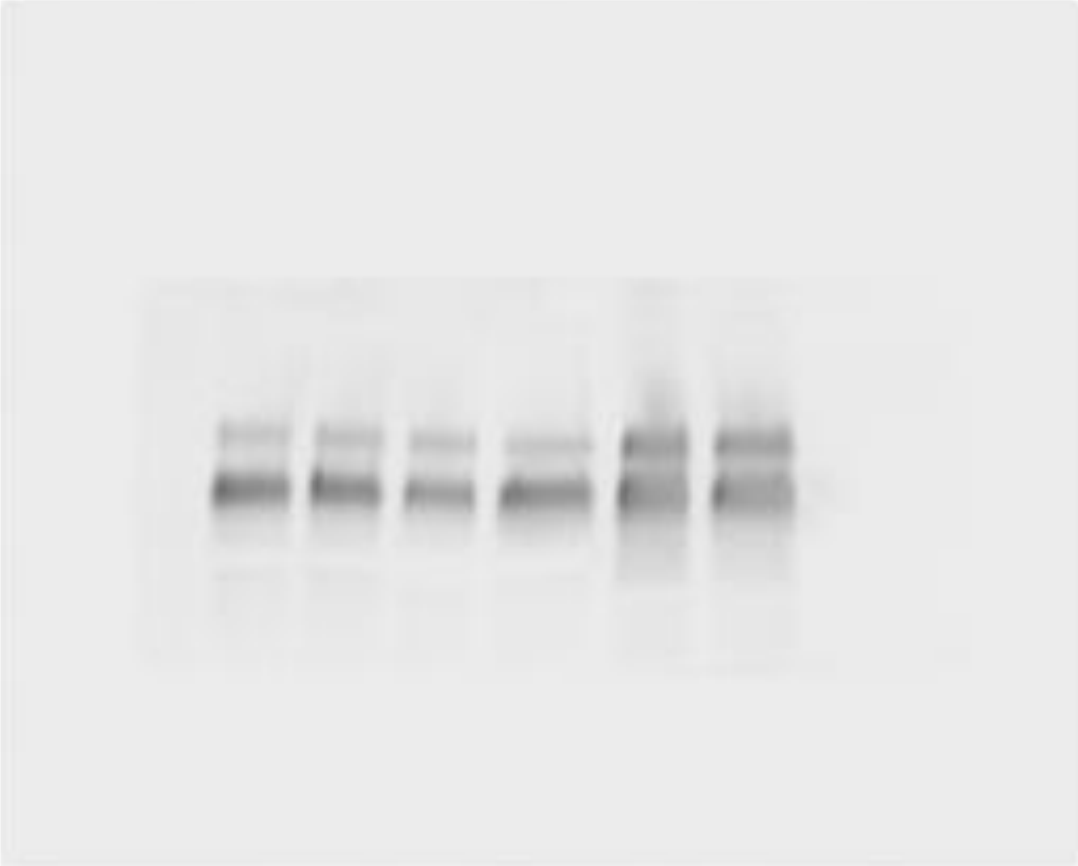

Supplementary Figure 3. pAKT

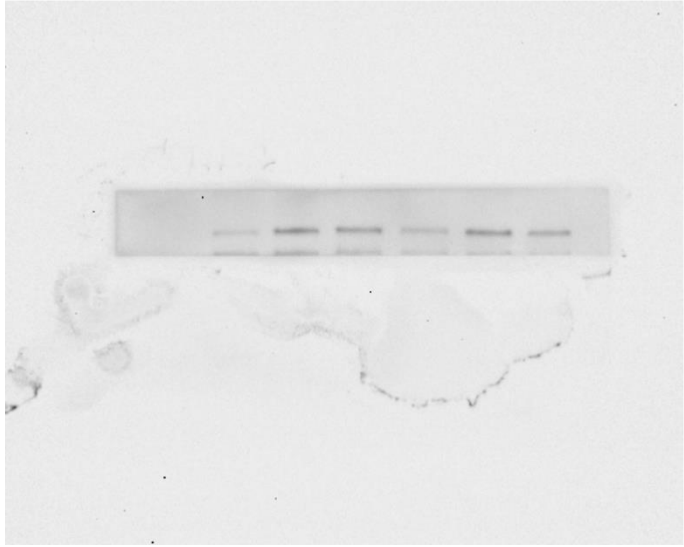

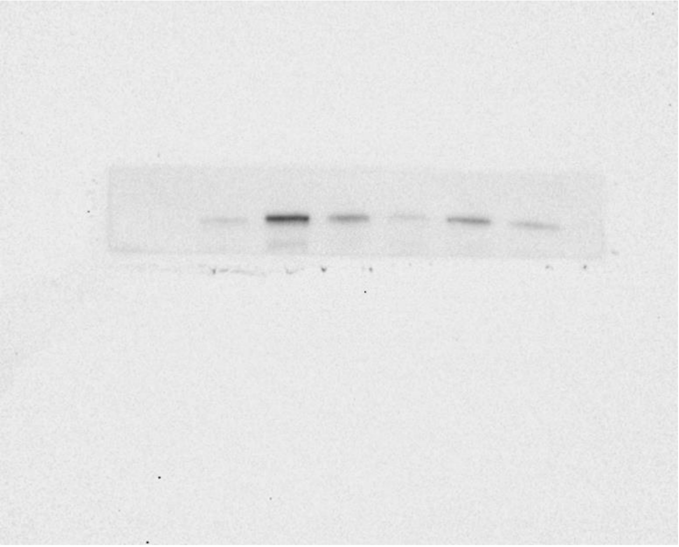

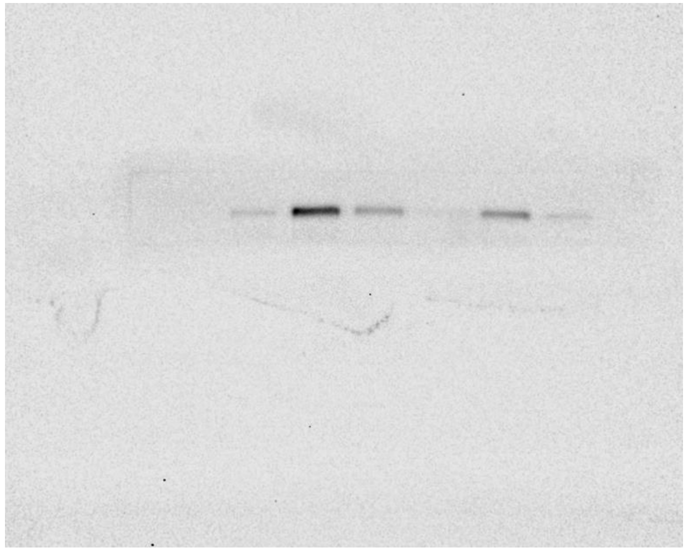

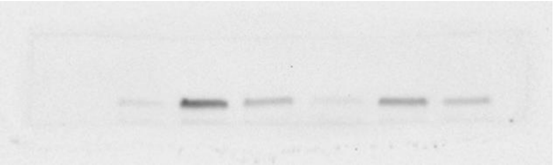

Supplementary Figure 3. pERK1/2

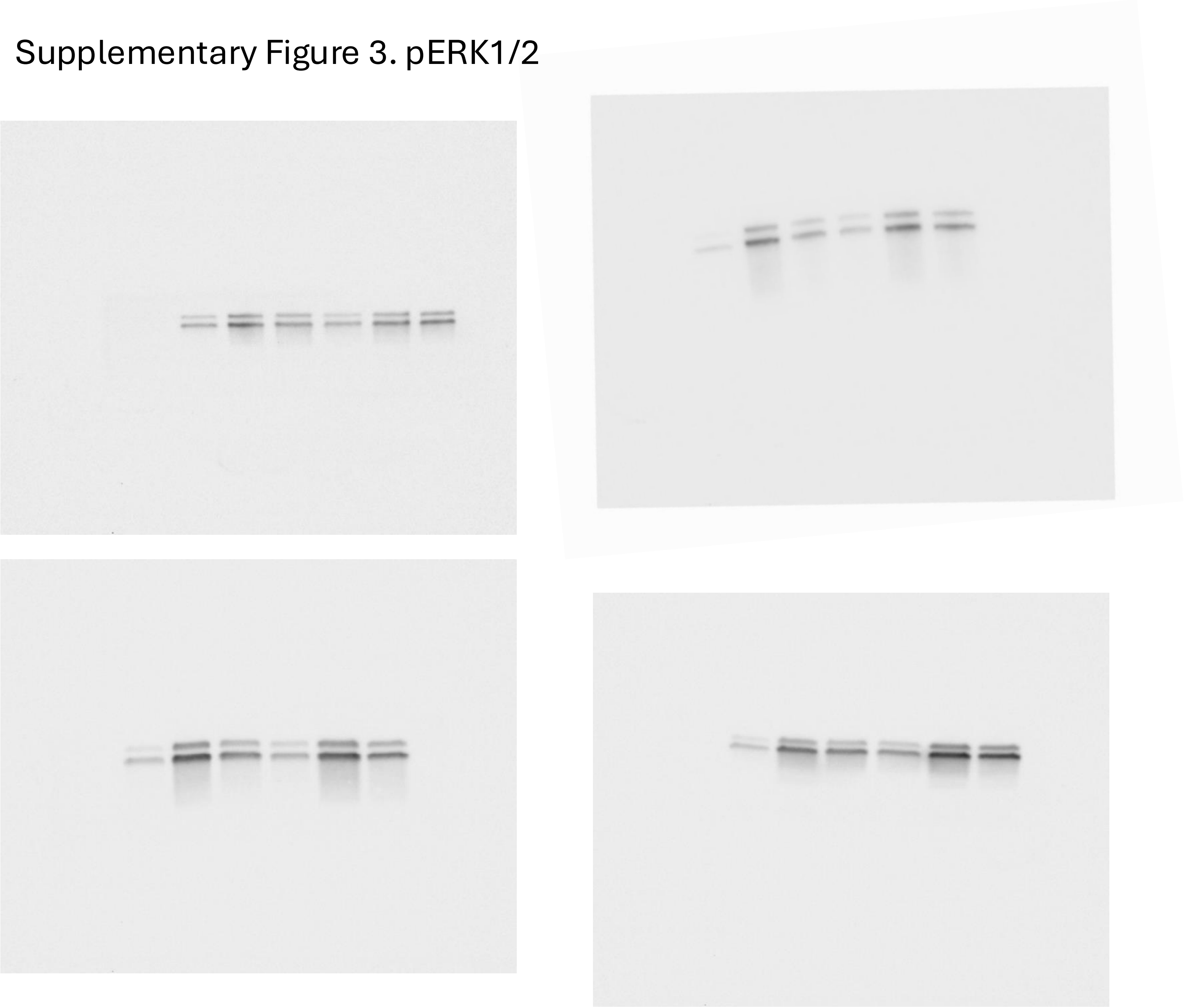

Supplementary Figure 3. total AKT

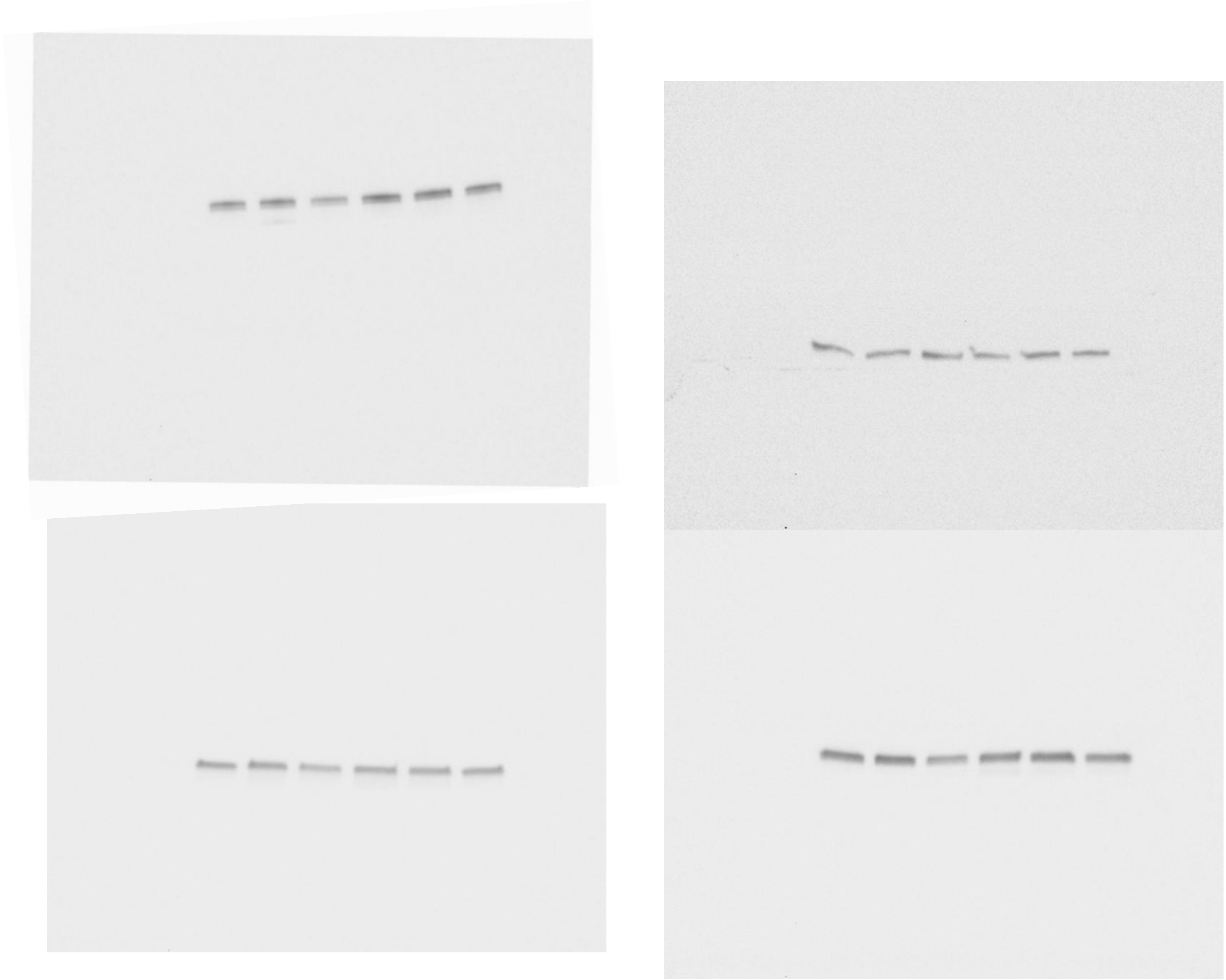

Supplementary Figure 3. total ERK1/2

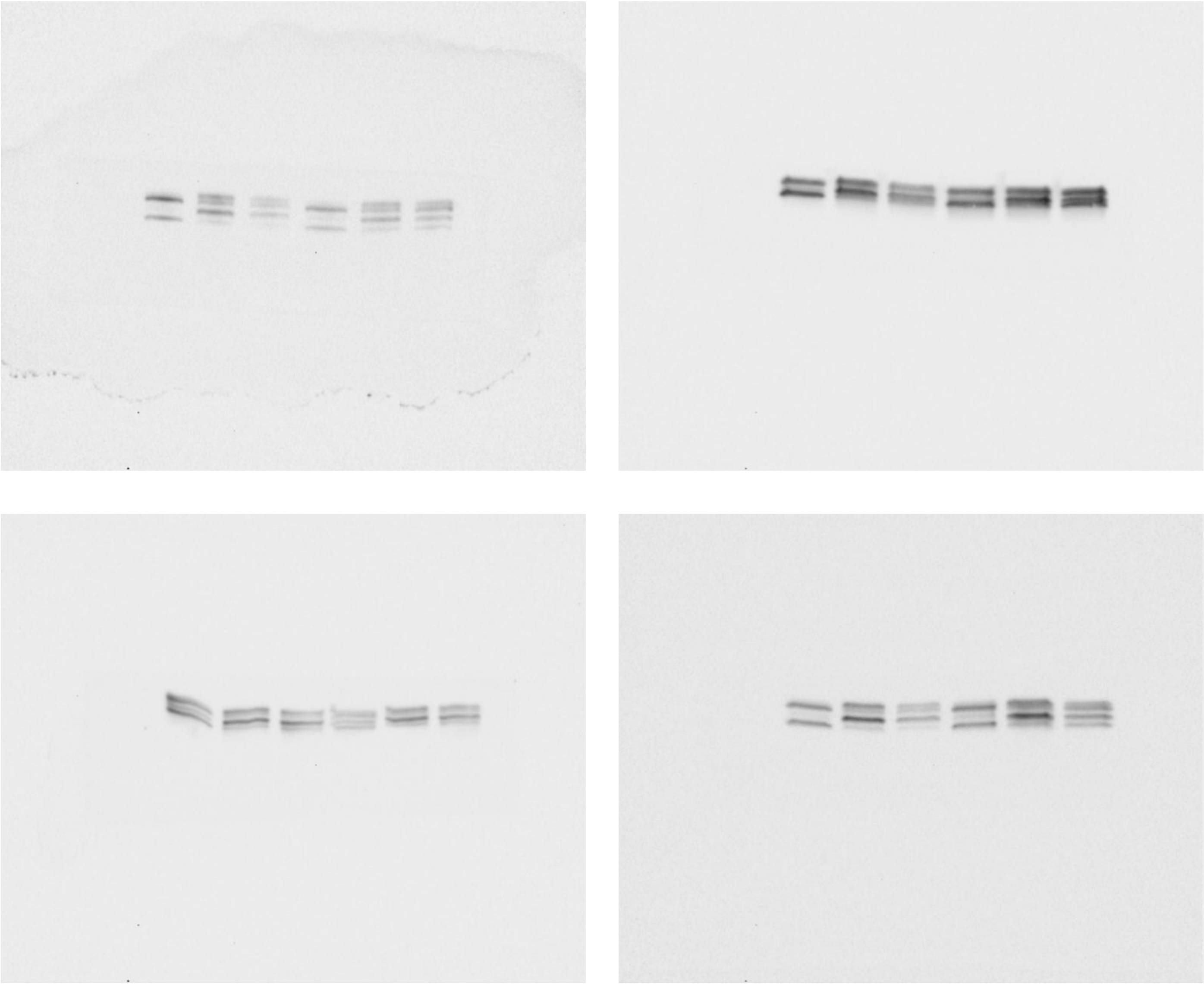

Supplementary Figure 3. phospho blot GAPDH

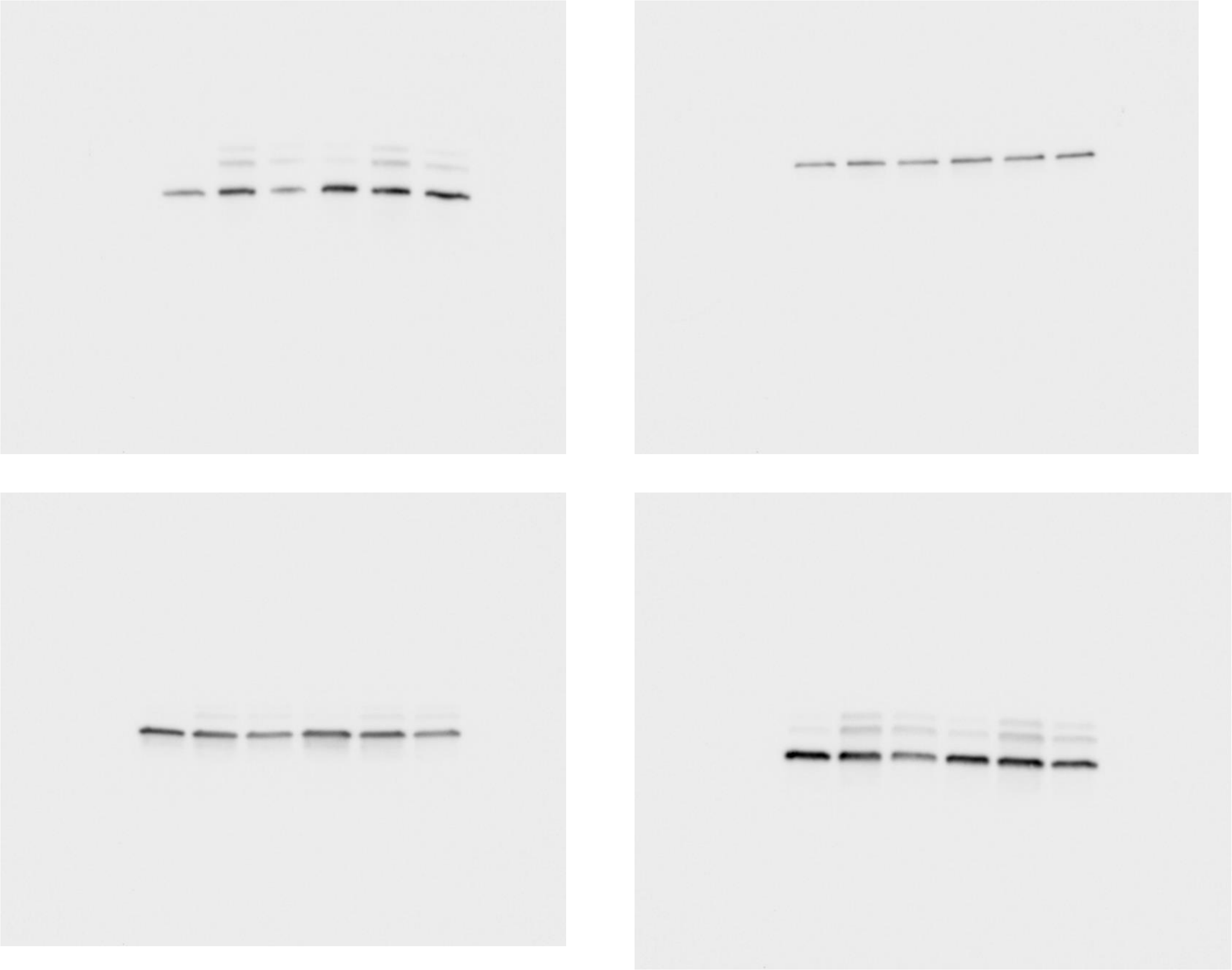

Supplementary Figure 3. total blot GAPDH

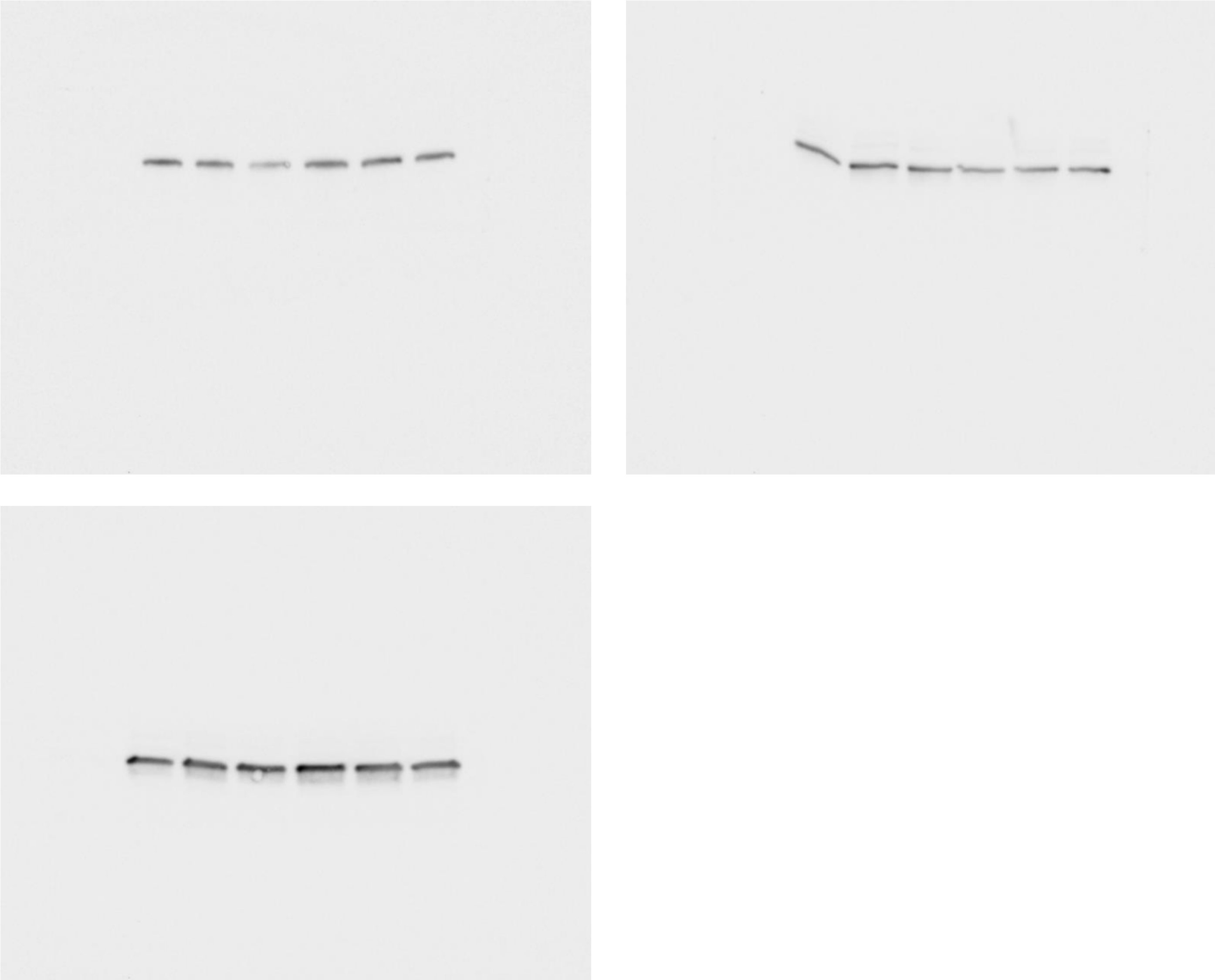

Supplementary Figure 4.

pERK1/2

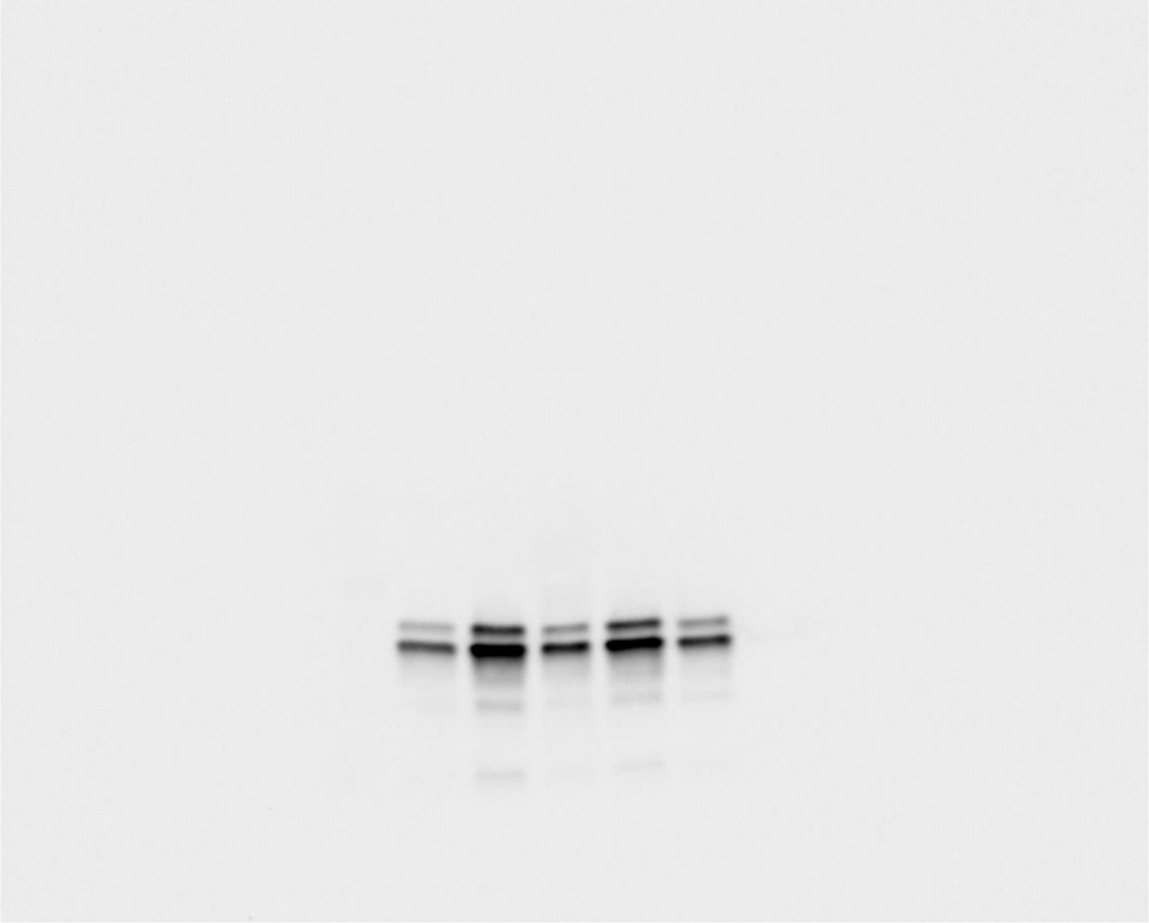

GAPDH

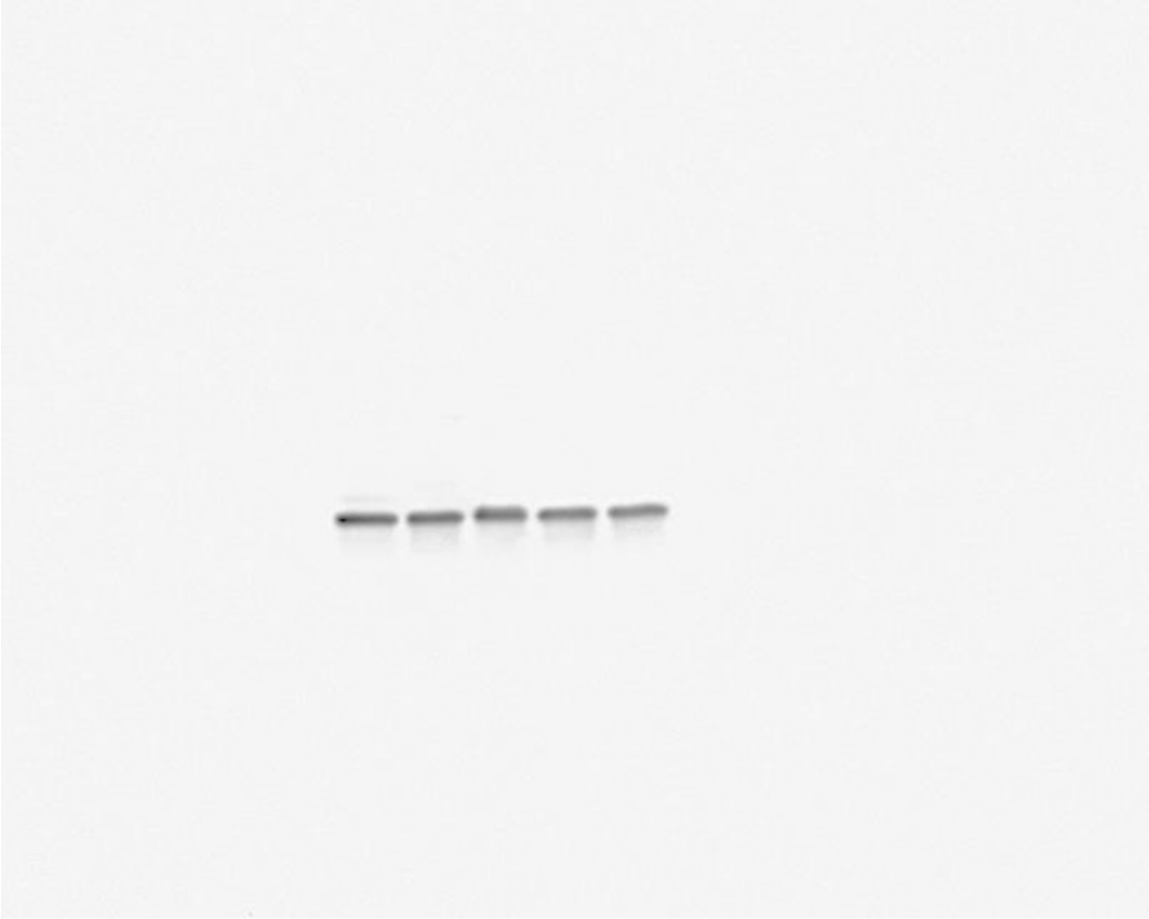

Supplementary Figure 4.

Total ERK1/2

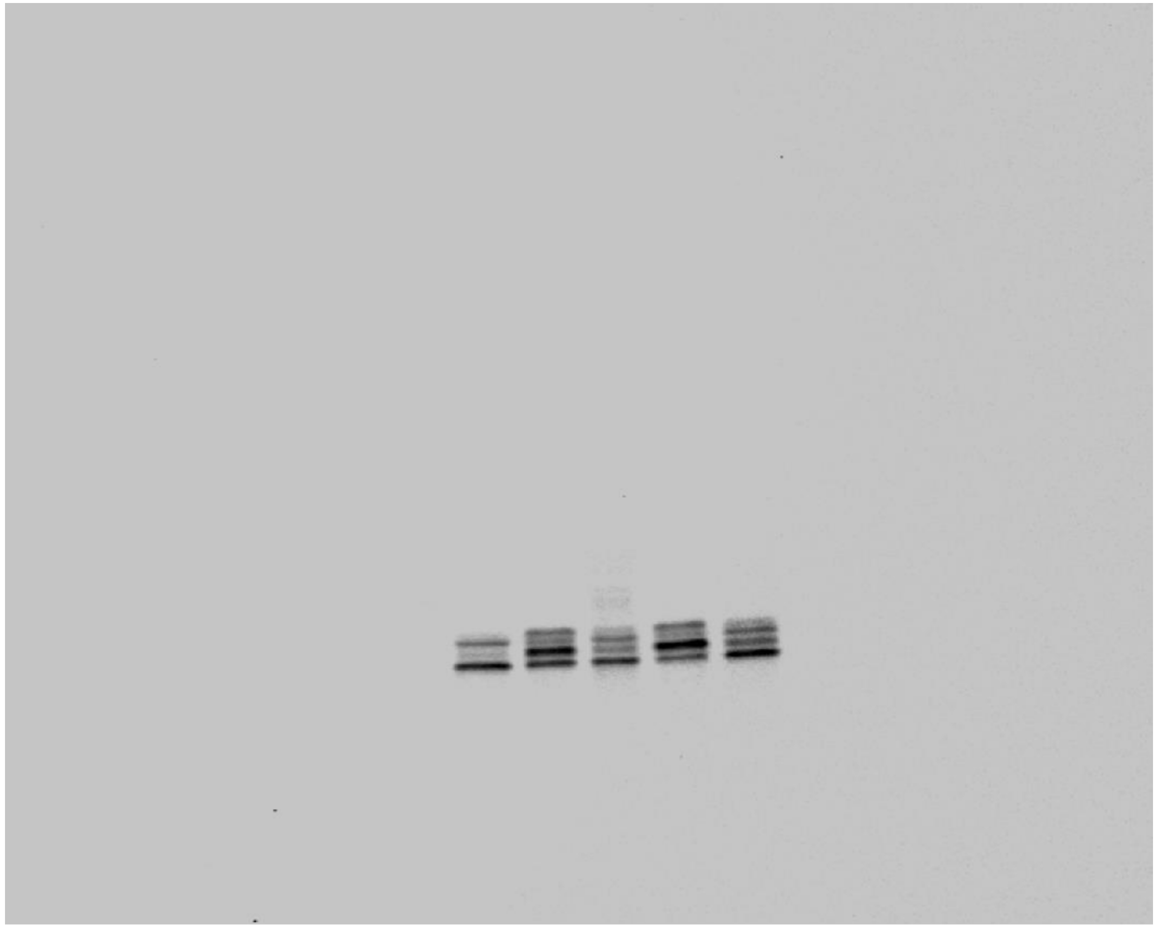

GAPDH

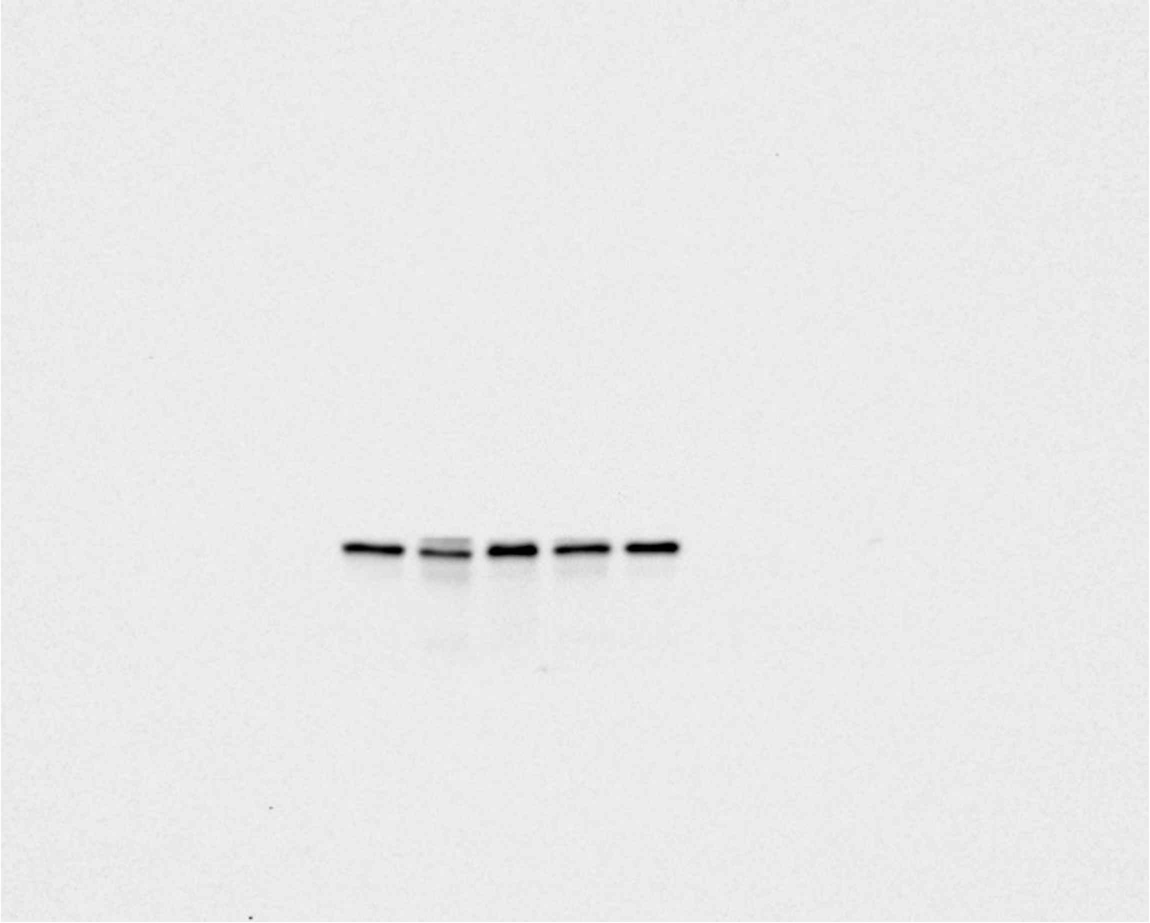

Supplement: Supplementary file 2 — Supplementary Western Blots [file 41419_2026_8464_MOESM2_ESM.pdf]
