# Supplementary figures and images for: Impaired BDNF-TrkB trafficking and signalling in Down syndrome basal forebrain neurons
Source: Cell Death Dis. 2026 Feb 11;17(1):214. doi: 10.1038/s41419-026-08464-z (PMC12921309; doi:10.1038/s41419-026-08464-z)

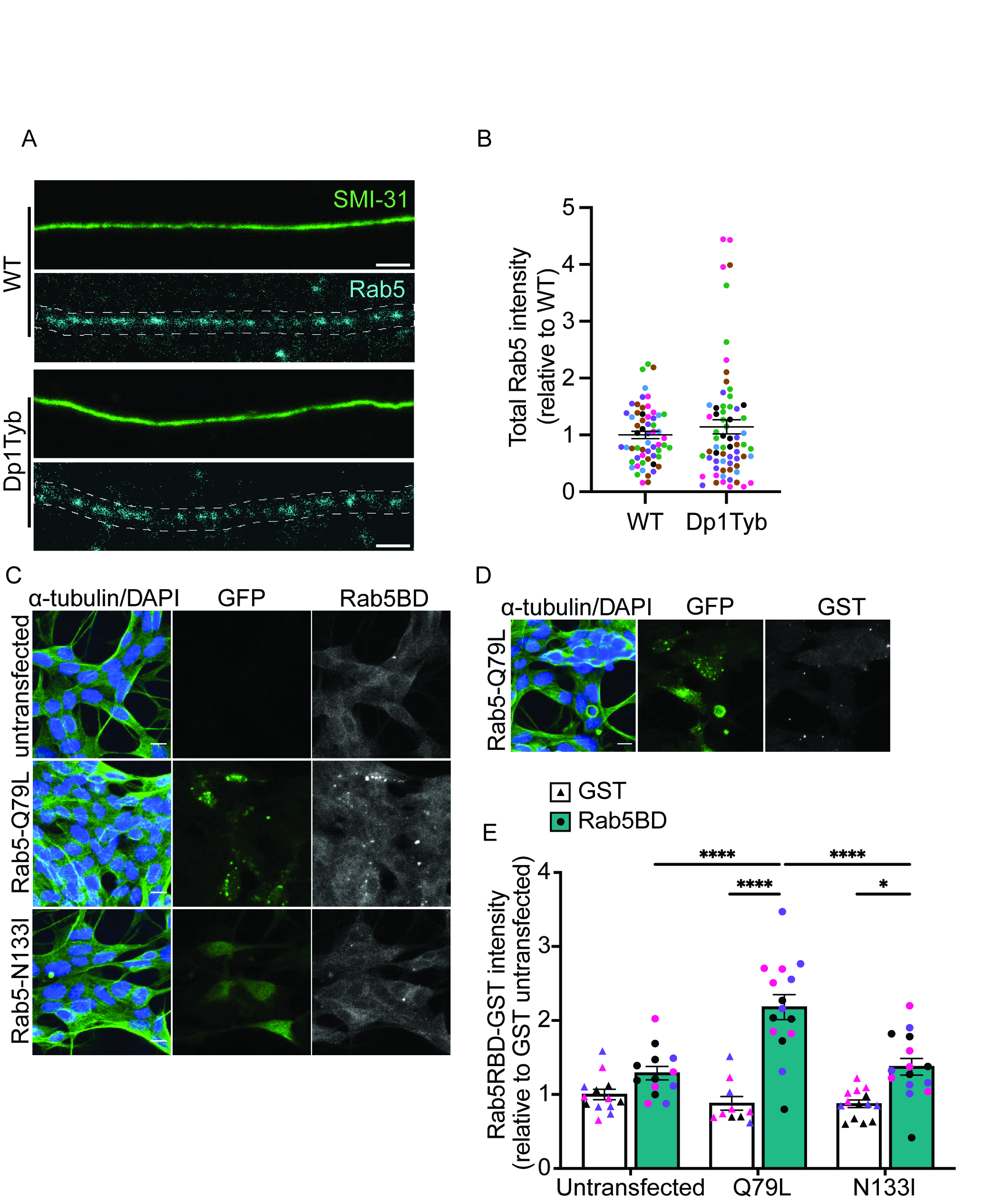

Supplement: Supplementary file 3 — Supplementary Figure 1 [file 41419_2026_8464_MOESM3_ESM.tif]

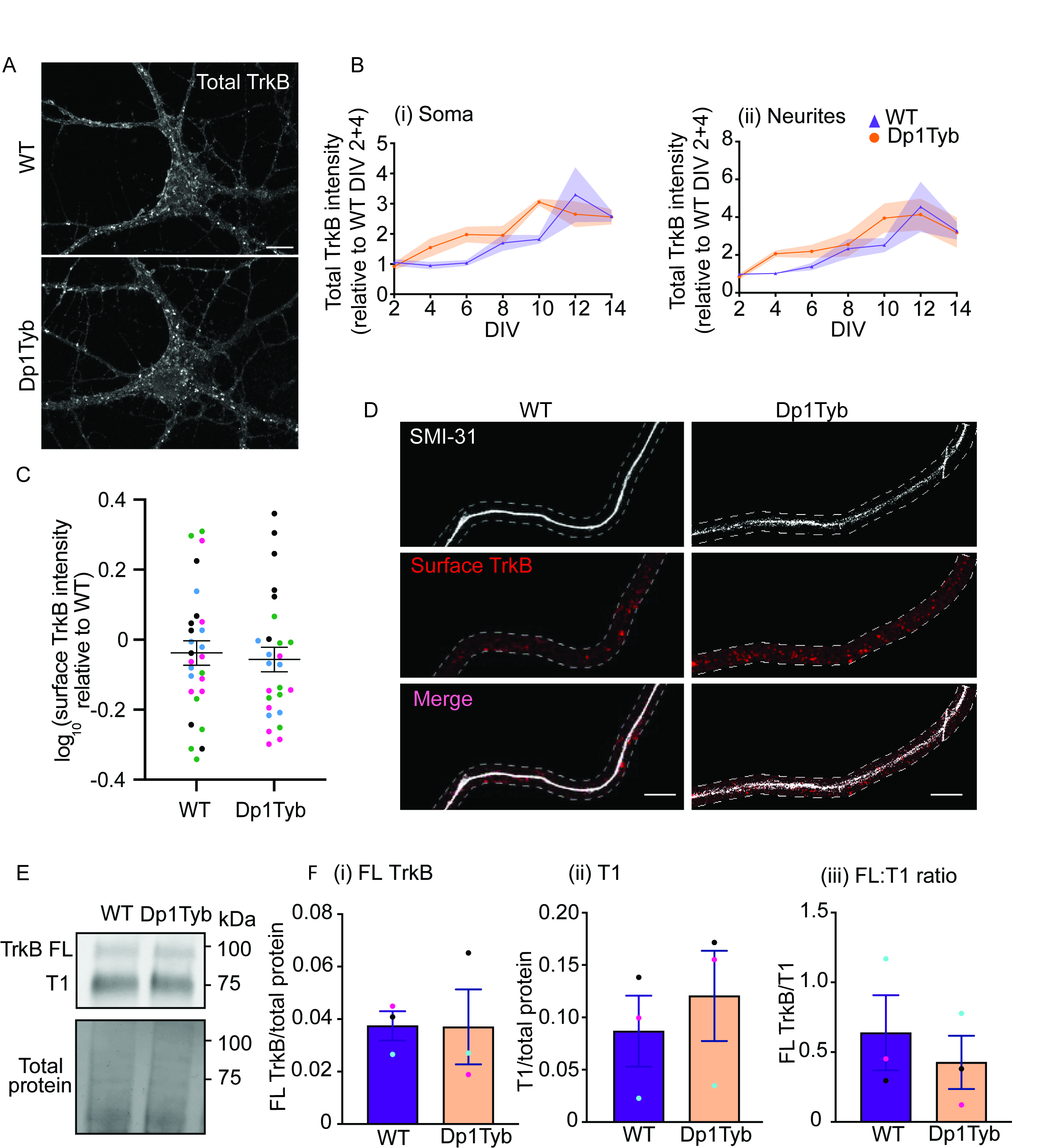

Supplement: Supplementary file 4 — Supplementary Figure 2 [file 41419_2026_8464_MOESM4_ESM.tif]

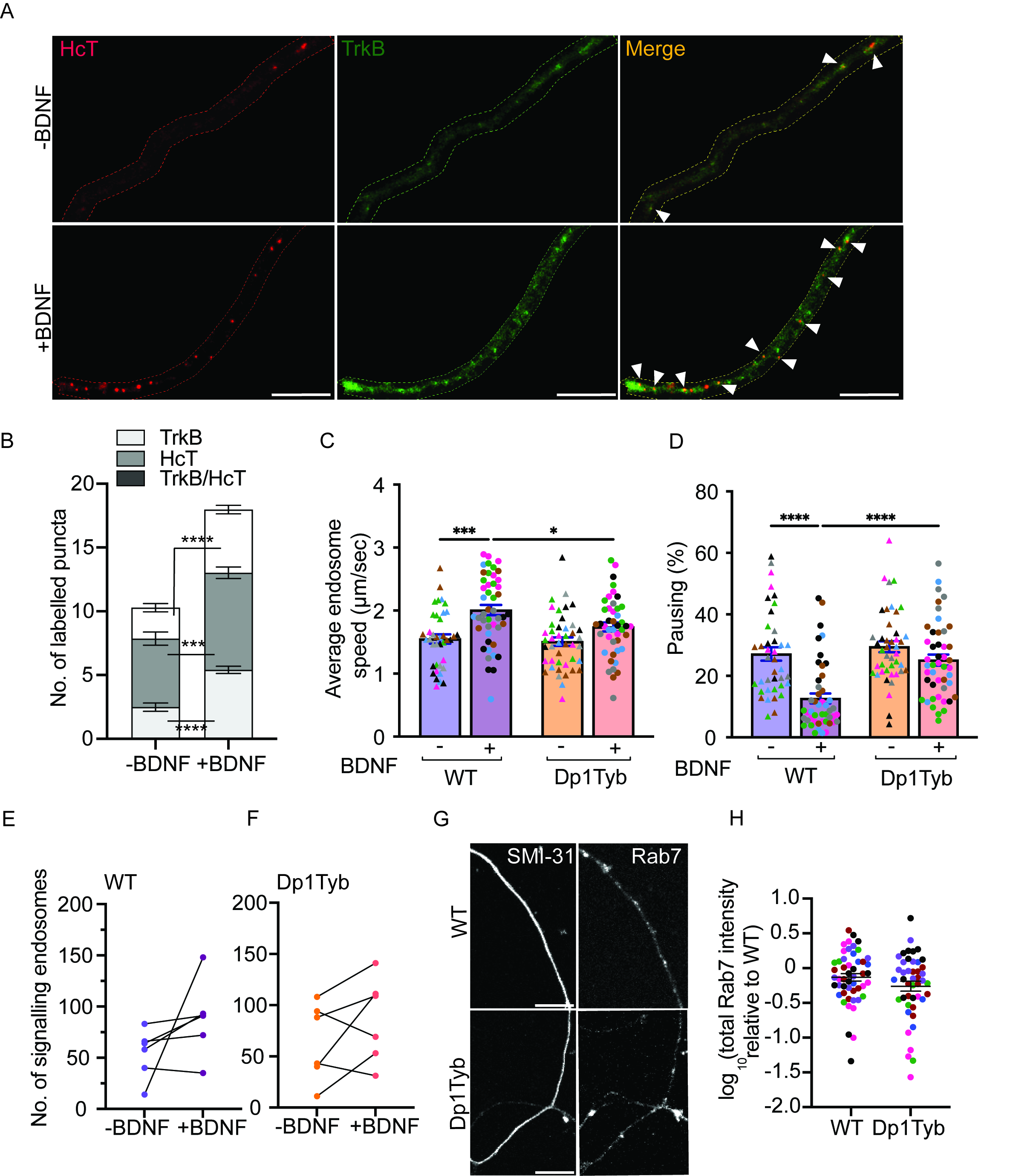

Supplement: Supplementary file 5 — Supplementary Figure 3 [file 41419_2026_8464_MOESM5_ESM.tif]

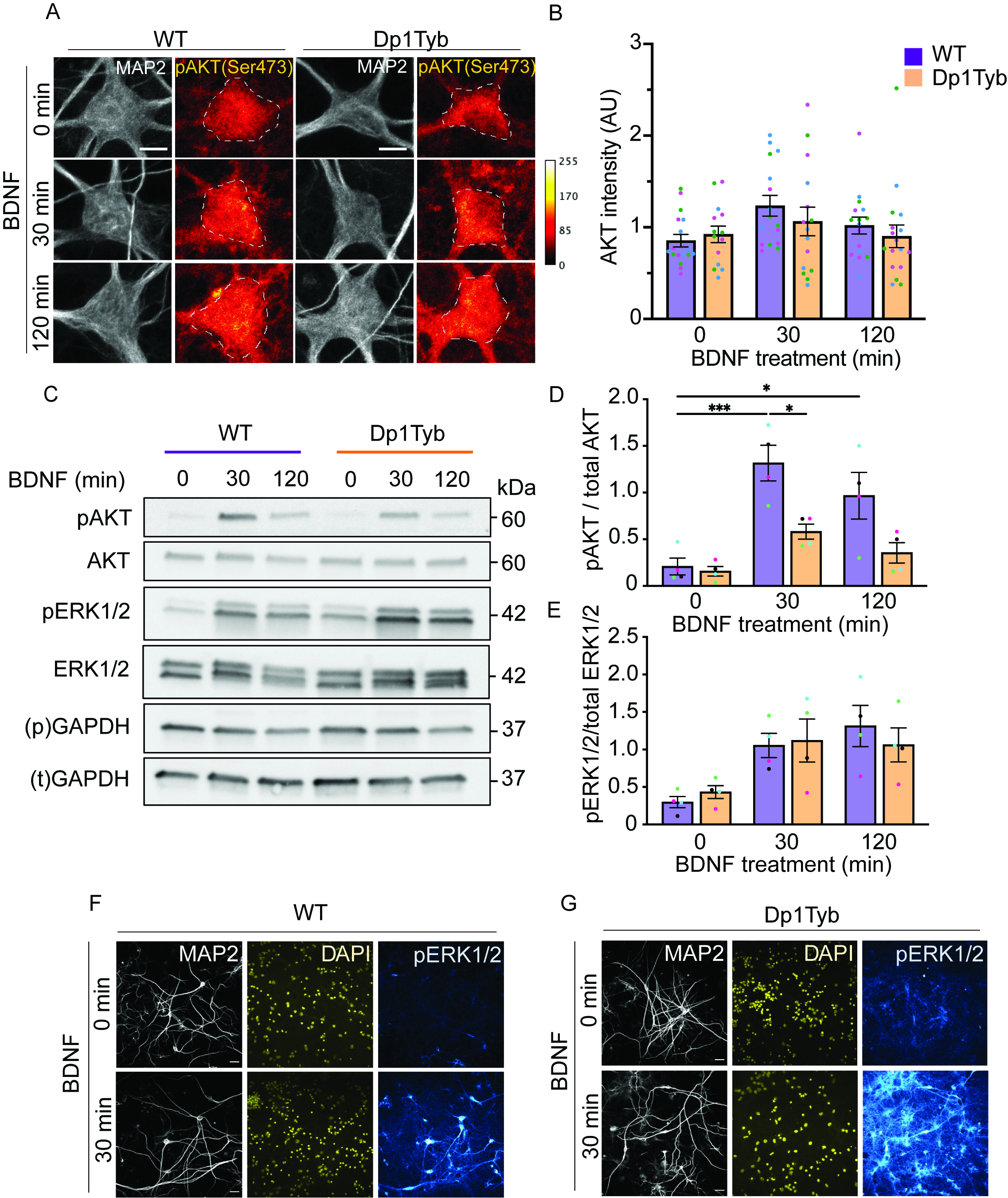

Supplement: Supplementary file 6 — Supplementary Figure 4 [file 41419_2026_8464_MOESM6_ESM.tif]

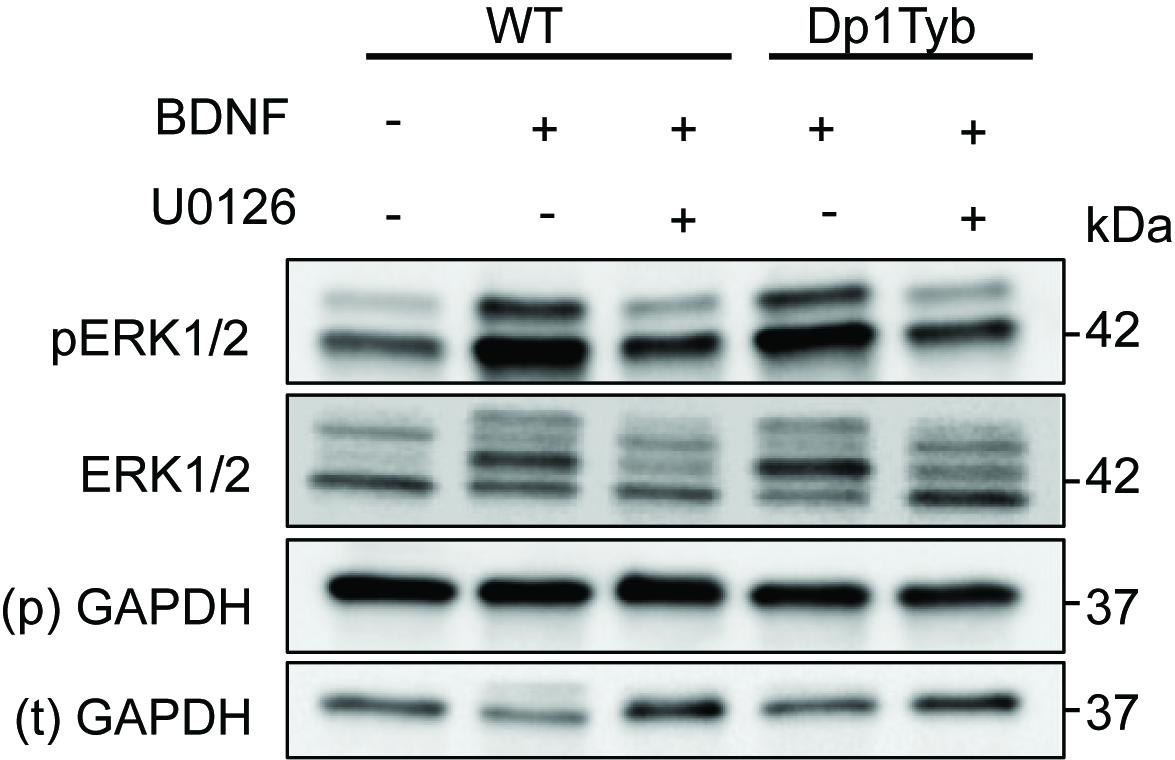

Supplement: Supplementary file 7 — Supplementary Figure 5 [file 41419_2026_8464_MOESM7_ESM.tif]
